# Effect of Atorvastatin Combined With Herb‐Partitioned Moxibustion on PON‐1 in Patients With Atherosclerotic Cerebral Infarction: A Randomized Controlled Trial

**DOI:** 10.1002/brb3.71565

**Published:** 2026-06-25

**Authors:** Wei An, Xinlei Hou, Ning Wei, Yuzhuo Zhou, Xinsheng Xie, Ze Jin, Linjing Wang, Xuzhou Li, Weijia Zhuang, Di Chen

**Affiliations:** ^1^ The Second Affiliated Hospital of Heilongjiang University of Traditional Chinese Medicine Harbin Heilongjiang China; ^2^ Heilongjiang University of Traditional Chinese Medicine Harbin Heilongjiang China

**Keywords:** atherosclerosis, atorvastatin, cerebral infarction, herb‐partitioned moxibustion, paraoxonase‐1, randomized controlled trial

## Abstract

**Background:**

Atherosclerotic cerebral infarction (ACI) is a major cause of disability and mortality worldwide. Atorvastatin provides lipid‐lowering and anti‐inflammatory benefits, whereas herb‐partitioned moxibustion (HPM) may improve circulation and vascular function. This study evaluated the effects of atorvastatin combined with HPM on oxidative stress, blood rheology, and vascular physiological function in patients with ACI.

**Methods:**

In this prospective randomized controlled trial, 100 patients with ACI admitted between December 2023 and October 2024 were randomly assigned to a research group receiving atorvastatin plus HPM (*n* = 50) or a control group receiving conventional Western medicine alone (*n* = 50). Treatment lasted 12 weeks. Primary outcomes were serum paraoxonase‐1 (PON‐1) and oxidized low‐density lipoprotein (ox‐LDL). Secondary outcomes included blood rheology indexes, vascular physiological function parameters, clinical efficacy, and adverse reactions.

**Results:**

All patients completed the study. Compared with the control group, the research group showed significantly lower whole blood viscosity at high shear (5.10 ± 1.23 vs. 6.15 ± 1.02 mPa·s), low shear (8.10 ± 1.17 vs. 10.46 ± 1.09 mPa·s), and plasma viscosity (1.20 ± 0.28 vs. 1.88 ± 0.36 mPa·s) (all *p* < 0.05). PON‐1 levels were higher (185.62 ± 56.28 vs. 162.20 ± 54.24 µg/L; *p* = 0.037) and ox‐LDL levels were lower (448.24 ± 80.64 vs. 500.40 ± 85.20 kU/L; *p* = 0.002). In addition, vascular physiological function improved significantly, with higher peak A, stroke volume, and ejection fraction and lower peak E and MVCF (all *p* < 0.001). The total effective rate was higher in the research group (94.0% vs. 82.0%; *p* = 0.037), with comparable adverse‐event rates.

**Conclusion:**

Atorvastatin combined with HPM improved oxidative stress, blood rheology, vascular physiological function, and clinical outcomes in ACI without increasing adverse reactions.

## Graphical abstract

Atorvastatin combined with herb‐partitioned moxibustion significantly improved oxidative stress markers, blood rheology, vascular physiological function, and overall clinical efficacy in patients with atherosclerotic cerebral infarction while maintaining a favorable safety profile.

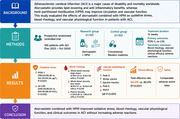

**Abbreviations**
ACI: atherosclerotic cerebral infarction
BMI: body mass index
CONSORT: Consolidated Standards of Reporting Trials
CT: computed tomography
EF: ejection fraction
ELISA: enzyme‐linked immunosorbent assay
HMG‐CoA: 3‐hydroxy‐3‐methylglutaryl‐coenzyme A
HPM: herb‐partitioned moxibustion
MRI: magnetic resonance imaging
MVCF: mean velocity of circumferential fiber shortening
NIHSS: National Institutes of Health Stroke Scale
ox‐LDL: oxidized low‐density lipoprotein
Peak A: peak atrial systolic flow velocity
Peak E: peak early diastolic flow velocity
PON‐1: Paraoxonase 1
RCT: randomized controlled trial
SV: stroke volume
TCM: traditional Chinese medicine
TOAST: Trial of Org 10172 in Acute Stroke Treatment

## 1 Introduction

Atherosclerosis is the main pathological factor leading to cerebrovascular diseases, and atherosclerotic cerebral infarction (ACI) refers to the occurrence of atherosclerosis in intracranial arteries (Gutierrez et al. 2022); it mainly manifests as vascular lumen stenosis or even occlusion, eventually leading to thrombus formation, which affects cerebral blood circulation and causes focal acute cerebral ischemia. Clinically, the major manifestations include limb weakness, abnormal sensation, speech disturbance, and cognitive impairment (Min et al. 2023; Wang et al. 2021). Clinical treatments mainly aim to improve the disease by reducing blood lipids, softening, or even shrinking intracranial atherosclerotic plaques (Xu et al. 2023). Among pharmacological interventions, statins have been established as the cornerstone therapy. Studies have found that statins possess significant lipid‐lowering effects and can improve the function of vascular endothelial cells, inhibit inflammatory reactions, suppress the proliferation of smooth muscle cells, promote cell apoptosis, reduce lipid deposition in vascular endothelial cells, decrease the formation of foam cells, and inhibit platelet activity and aggregation to stabilize atherosclerotic plaques (Chen et al. 2020; Fujisue et al. 2021; Harm et al. 2023).

Herb‐partitioned moxibustion (HPM) is a form of modern indirect moxibustion that integrates the dual therapeutic advantages of moxibustion heat stimulation and herbal pharmacology (Yang et al. 2024). In this technique, a herbal medicinal cake composed of selected traditional Chinese medicine ingredients is placed on specific acupoints, and a moxa cone is ignited on top of the cake. The burning moxa generates thermal radiation that penetrates the skin surface, while the medicinal components of the herbal cake are simultaneously activated by the heat and absorbed transdermally. Through this combined thermal–pharmacological action, HPM promotes blood circulation, resolves blood stasis, warms the meridians, and expels cold. Within the framework of traditional Chinese medicine theory, ACI belongs to the categories of “stroke,” “dizziness,” “forgetfulness,” “dementia,” “pulse obstruction,” and “jue syndrome,” which are primarily attributed to blood stasis syndrome. According to TCM pathogenesis, qi deficiency and blood stasis lead to obstruction of the brain collaterals, and HPM targets this pathological mechanism by invigorating qi, activating blood flow, unblocking collateral vessels, and restoring cerebral perfusion (Zhang et al. 2023; Deng and Li 2023). Modern pharmacological research has demonstrated that moxibustion exerts its therapeutic effects partly by inhibiting Ca^2^^+^ overload‐triggered oxidative stress and modulating inflammatory responses via the P2Y12/PI3K/AKT signaling pathway (Yang et al. 2024). Furthermore, the herbal ingredients used in the medicinal cake, derived from the classical formulas Shaoyao Licorice Decoction and Buyang Huanwu Decoction, are known to improve microcirculation, reduce blood viscosity, and exert neuroprotective effects (Wang et al. 2022; Xuan et al. 2023).

The rationale for combining atorvastatin with HPM lies in their complementary mechanisms of action. Atorvastatin primarily targets systemic lipid metabolism and vascular inflammation through inhibition of HMG‐CoA reductase, while HPM acts through local acupoint stimulation and transdermal herbal absorption to promote blood circulation and regulate the qi‐blood balance. This combination therefore addresses ACI through both the macro‐level reduction of atherosclerotic risk factors and the micro‐level improvement of local cerebrovascular hemodynamics. Although there are currently many studies on combined treatment of traditional Chinese and Western medicine for cerebrovascular diseases, the results are inconsistent, and no study has specifically analyzed the clinical efficacy of atorvastatin combined with HPM for ACI, particularly regarding the effects on PON‐1 and ox‐LDL as markers of oxidative stress. Therefore, this randomized controlled trial was designed to evaluate the therapeutic effect of atorvastatin combined with HPM in patients with ACI, focusing on changes in PON‐1, ox‐LDL, blood rheology, and vascular physiological function.

## 2 Materials and Methods

### 2.1 Study Design

This was a prospective, single‐center, parallel‐group, randomized controlled trial (RCT) conducted at the Second Affiliated Hospital of Heilongjiang University of Traditional Chinese Medicine from December 2023 to October 2024. The study protocol was approved by the institutional ethics committee (no. IRB‐AF/SG‐21/02.0), and all enrolled patients provided written informed consent. The trial was conducted and reported in accordance with the Consolidated Standards of Reporting Trials (CONSORT) guidelines (Schulz et al. 2010), and a completed CONSORT checklist is provided as a Supplementary File.

### 2.2 Sample Size Estimation

The sample size was calculated based on the primary outcome of serum PON‐1 levels after treatment. Based on prior literature (Ferretti et al. 2015; Huang et al. 2013), a clinically meaningful between‐group difference in PON‐1 of 23 µg/L was assumed, with a pooled standard deviation of 55 µg/L. With a two‐sided significance level of *α* = 0.05 and a statistical power of 80% (1 – *β* = 0.80), the minimum required sample size was calculated as 45 patients per group using the formula *n* = 2(*Z* ^α/2^ + *Z* ^β^)^2^ *σ* ^2^/*δ* ^2^. Accounting for a potential 10% dropout rate, a total of 100 patients (50 per group) were enrolled.

### 2.3 Participants

During the study period, a total of 136 consecutive inpatients with suspected ACI were screened for eligibility. Of these, 26 patients were excluded because they did not meet the inclusion criteria (*n* = 14), met one or more exclusion criteria (*n* = 8), or declined to participate (*n* = 4). The remaining 110 eligible patients were assessed, and 100 were enrolled and randomized. All patients were hospitalized during the initial treatment period and subsequently continued treatment on an outpatient basis with scheduled follow‐up visits at weeks 4, 8, and 12.

Inclusion criteria were as follows: (1) meeting the diagnostic criteria for cerebral infarction established by the Fourth National Cerebrovascular Disease Academic Conference in 1995; (2) confirmation by head computed tomography (CT) or magnetic resonance imaging (MRI) examination; (3) classification as atherosclerotic thrombosis according to the modified TOAST classification (Han et al. 2006); (4) ultrasound examination demonstrating atherosclerotic plaque formation in the carotid arteries; (5) onset time ≤ 24 h from symptom presentation.

Exclusion criteria included: (1) history of prior stroke or pre‐existing neurological disease; (2) malignant tumors; (3) active infectious diseases; (4) severe heart, liver, or kidney dysfunction; (5) known systemic inflammatory conditions or autoimmune diseases that could confound inflammatory marker assessment; (6) concurrent use of other statins, anticoagulants, or traditional Chinese medicine treatments within the preceding 4 weeks.

### 2.4 Randomization and Allocation

Eligible patients were randomly assigned in a 1:1 ratio to the research group or the control group using a computer‐generated random number table. The randomization sequence was generated by an independent statistician who was not involved in patient recruitment or treatment. Sequentially numbered, opaque, sealed envelopes containing group assignments were prepared in advance. Upon enrollment, each patient was assigned the next sequential envelope. Due to the nature of the intervention (moxibustion), blinding of patients and treating physicians was not feasible; however, the outcome assessors and the statistician performing the data analysis were blinded to group allocation.

### 2.5 Interventions

Patients in the control group received standardized conventional Western medicine treatment, which included aspirin enteric‐coated tablets (Bayer HealthCare, 100 mg/day, orally) for antiplatelet therapy, and citicoline sodium injection (0.5 g dissolved in 250 mL of 0.9% sodium chloride, administered intravenously once daily for the initial 14 days, followed by citicoline sodium tablets 200 mg orally three times daily) for cerebral circulation improvement. Supportive care, including blood pressure management, blood glucose control, and nutritional support, was provided as clinically indicated.

Patients in the research group received all treatments identical to the control group, plus atorvastatin calcium tablets (Pfizer Inc., National Drug Approval Number: H20203133, 10 mg/tablet) administered orally at a dose of 20 mg once daily, and HPM. The herbal medicinal cake was prepared from a combination of Shaoyao Licorice Decoction and Buyang Huanwu Decoction, ground into fine powder and mixed with rice vinegar to form a paste. The paste was shaped into circular cakes approximately 3 cm in diameter and 0.5 cm in thickness. For each treatment session, the patient was positioned in a prone or supine posture depending on the target acupoints. The acupoints were identified, the surrounding skin was cleaned and disinfected, and the prepared medicinal cake was placed on the identified acupoints (Baihui, Zusanli, Quchi, and Xuehai). A moxa cone was lit and placed on top of each medicinal cake. When the moxa cone burned out and all residual heat dissipated, a second moxa cone was applied. Approximately 3 to 4 moxa cones were used per acupoint per session (approximately 20 to 30 min total per session). HPM was performed once daily throughout the 12‐week treatment period. Both groups followed an identical follow‐up schedule, with assessments conducted at baseline, week 4, week 8, and week 12.

### 2.6 Baseline Assessment and Disease Severity

At enrollment, the following baseline variables were collected for all patients: age, sex, body mass index (BMI), medical history (hypertension, diabetes mellitus, hyperlipidemia, smoking history), time from symptom onset to hospital admission, and National Institutes of Health Stroke Scale (NIHSS) score to assess baseline neurological deficit severity. All patients underwent head CT at admission. Baseline disease severity was assessed using the NIHSS, and comparability between the two groups was confirmed. The mean NIHSS score at baseline was 8.62 ± 3.14 in the research group and 8.88 ± 3.26 in the control group, with no statistically significant difference (*t* = 0.406, *p* = 0.686). Additionally, the distributions of vascular risk factors including hypertension (research group: 64.00% vs. control group: 60.00%, *χ* ^2^ = 0.170, *p* = 0.680), diabetes mellitus (22.00% vs. 26.00%, *χ* ^2^ = 0.219, *p* = 0.640), and smoking history (38.00% vs. 34.00%, *χ* ^2^ = 0.174, *p* = 0.677) were comparable between the two groups. Head CT confirmed the diagnosis of acute cerebral infarction in all enrolled patients. Regarding the extent of atherosclerosis in other arterial territories, all patients underwent carotid duplex ultrasound at baseline, which was an inclusion criterion. Additionally, 78 patients (78.00%) underwent CT angiography of the head and neck. Among the enrolled patients, carotid artery plaques were present in all 100 patients (100.00%), intracranial arterial stenosis was identified in 68 patients (68.00%), and peripheral arterial disease was documented in 12 patients (12.00%). The distribution of multi‐territory atherosclerotic involvement did not differ significantly between the two groups (*χ* ^2^ = 0.291, *p* = 0.589). Although concomitant atherosclerosis in other vascular beds may influence systemic inflammatory markers, both groups demonstrated comparable baseline levels of atherosclerotic burden, thereby minimizing the potential confounding effect on the between‐group comparisons of inflammatory and oxidative stress markers.

### 2.7 Specimen Collection

Blood specimens were collected at two time points: baseline (day 0, defined as the morning of the day before the first treatment session) and post‐treatment (day 84 ± 2, defined as the morning after the completion of the 12‐week treatment course). At each time point, 5 to 10 mL of fasting antecubital venous blood was drawn from each patient between 7:00 and 9:00 AM. The blood samples were centrifuged at 3000 r/min for 10 min at room temperature with a centrifugal radius of 10 cm, and the supernatant serum was separated and stored at −80°C until analysis.

### 2.8 Outcome Measures

The primary outcome measures were serum PON‐1 and ox‐LDL levels, as these are key biomarkers reflecting oxidative stress status in atherosclerotic disease. Secondary outcome measures included (1) blood rheology indexes (whole blood viscosity at high shear, whole blood viscosity at low shear, and blood viscosity), measured using a fully automatic hemorheology detector; (2) cerebrovascular physiological function parameters measured by color Doppler flow imaging, including maximum atrial systolic flow velocity of mitral valve blood flow (peak A), maximum early diastolic flow velocity of mitral valve blood flow (peak E), stroke volume (SV), left ventricular ejection fraction (EF), and mean velocity of circumferential fiber shortening (MVCF); (3) clinical efficacy evaluation, defined as follows: recovery was defined as the complete disappearance of dizziness and other symptoms with no positive signs on examination; markedly effective was defined as the absence of dizziness with only mild instability upon sitting up; effective was defined as occasional episodes of dizziness with a significantly reduced frequency of attacks; ineffective was defined as no obvious improvement in symptoms and signs. The total effective rate was calculated as (recovery + markedly effective + effective)/total × 100%; (4) adverse reactions including gastrointestinal reactions, allergic reactions, and circulatory system reactions.

### 2.9 Statistical Methods

SPSS 22.0 statistical software (IBM Corp., Armonk, NY, USA) was used for data processing and statistical analysis. Continuous data were tested for normality using the Shapiro–Wilk test. Measurement data conforming to normal distribution were expressed as mean ± standard deviation (¯*x* ± s), and independent‐samples *t*‐test was used for between‐group comparisons. Paired‐samples *t*‐test was used for within‐group pre‐ and post‐treatment comparisons. Enumeration data were expressed as frequency and percentage [*n* (%)], and the chi‐squared (*χ* ^2^) test was used for comparisons. A two‐sided *p* < 0.05 was considered statistically significant.

## 3 Results

### 3.1 Baseline Clinical Characteristics of the Patients

A total of 100 patients were enrolled and randomized, with 50 in each group. All 100 patients completed the full 12‐week treatment, and no patients were lost to follow‐up. In the comparison of baseline clinical characteristics including age [(62.50 ± 10.40) vs. (63.20 ± 11.60) years], sex distribution [male: 58.00% vs. 52.00%], BMI [(29.80 ± 5.56) vs. (30.40 ± 5.72) kg/m^2^], NIHSS score [(8.62 ± 3.14) vs. (8.88 ± 3.26)], hypertension prevalence [64.00% vs. 60.00%], and diabetes prevalence [22.00% vs. 26.00%] between the two groups, no statistically significant differences were observed (all *p* > 0.05), confirming that the groups were comparable at baseline (Table 1).

**TABLE 1:** Comparison of baseline clinical characteristics between the two groups.

Group	Age (years)	Gender		BMI (kg/m^2^)	NIHSS	Hypertension	Diabetes
		Male	Female			*n* (%)	*n* (%)
Research (*n* = 50)	62.50 ± 10.40	29(58.00)	21(42.00)	29.80 ± 5.56	8.62 ± 3.14	32(64.00)	11(22.00)
Control (*n* = 50)	63.20 ± 11.60	26(52.00)	24(48.00)	30.40 ± 5.72	8.88 ± 3.26	30(60.00)	13(26.00)
*t/χ* ^2^	0.318	0.364		0.530	0.406	0.170	0.219
*p*	0.751	0.546		0.597	0.686	0.680	0.640

### 3.2 Blood Rheology Index Levels of the Two Groups

**FIGURE 1:**
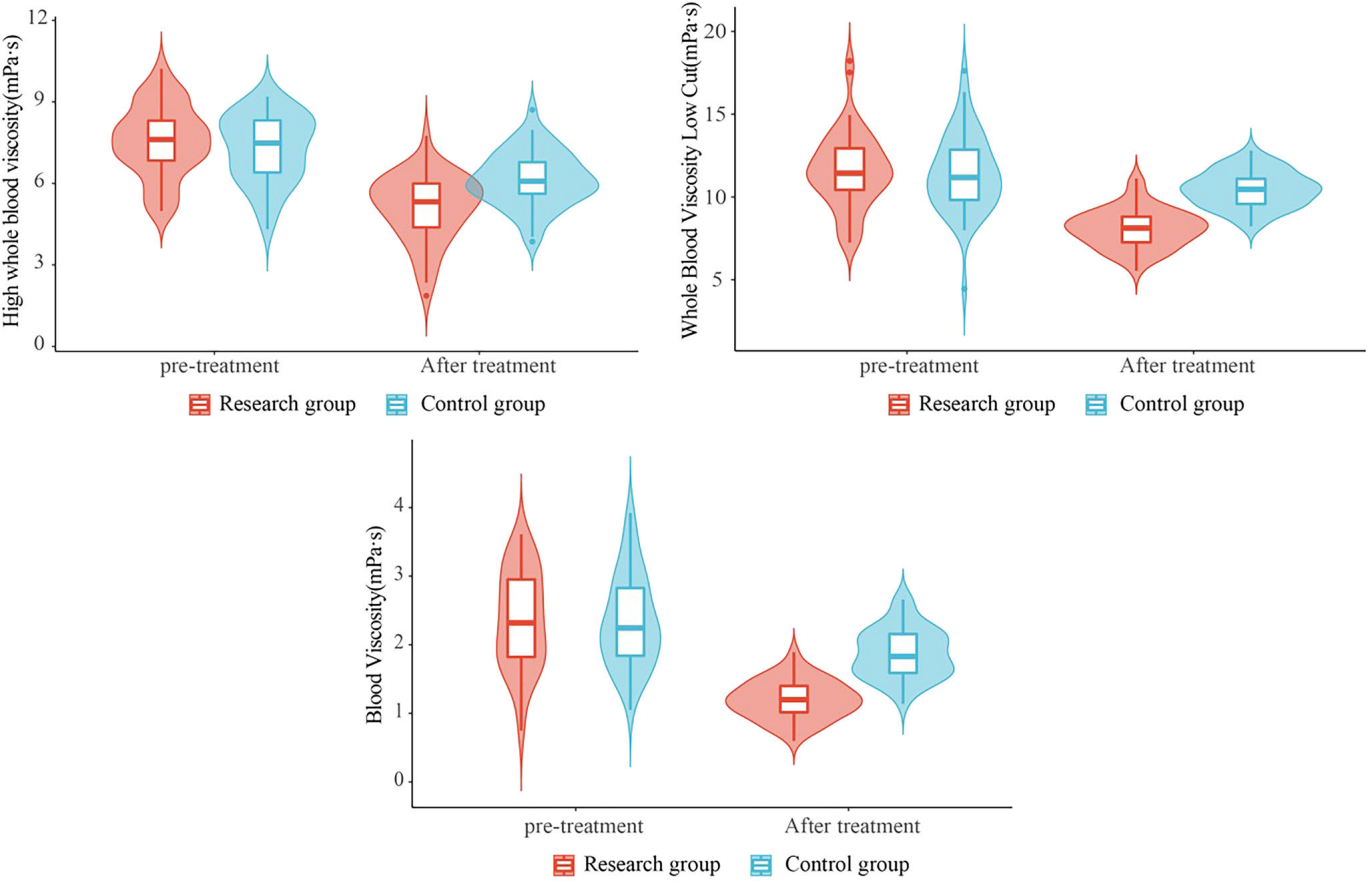
Comparison of blood rheology index levels between the two groups before and after therapy.

There was no statistically significant difference in blood rheology index levels between the two groups before therapy (*p* > 0.05). After therapy, compared with the control group [(6.15 ± 1.02), (10.46 ± 1.09), (1.88 ± 0.36) mPa·s], the blood rheology indexes of the research group [(5.10 ± 1.23), (8.10 ± 1.17), (1.20 ± 0.28) mPa·s] were significantly lower (all *p* < 0.001), as shown in Table 2 and Figure 1.

**TABLE 2:** Comparison of blood rheology index levels between the two groups before and after therapy (¯*x* ± s, mPa·s).

Group	Whole blood viscosity high shear		Whole blood viscosity low shear		Blood viscosity	
	Before	After	Before	After	Before	After
Research (*n* = 50)	7.57 ± 1.32	5.10 ± 1.23	11.58 ± 2.29	8.10 ± 1.17	2.36 ± 0.71	1.20 ± 0.28
Control (*n* = 50)	7.28 ± 1.25	6.15 ± 1.02	11.41 ± 2.36	10.46 ± 1.09	2.34 ± 0.67	1.88 ± 0.36
*t*	1.113	4.653	0.352	10.422	0.167	10.534
*p*	0.268	<0.001	0.726	<0.001	0.868	<0.001

### 3.3 PON‐1 and Ox‐LDL Levels of the Two Groups

**FIGURE 2:**
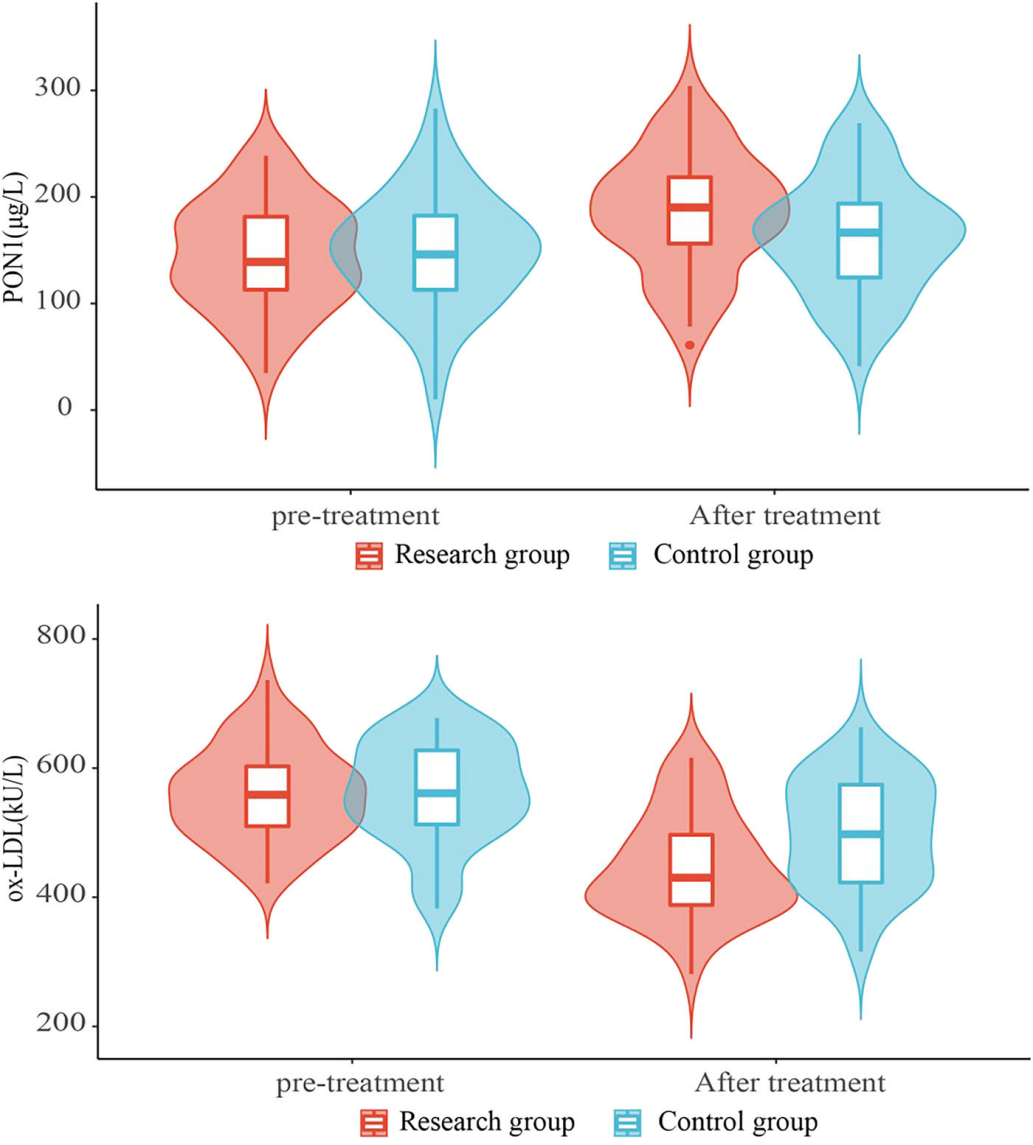
Comparison of PON‐1 and ox‐LDL levels between the two groups before and after therapy.

There was no statistically significant difference in PON‐1 and ox‐LDL levels between the two groups before therapy (*p* > 0.05). After therapy, compared with the control group [(162.20 ± 54.24) µg/L, (500.40 ± 85.20) kU/L], the research group demonstrated significantly higher PON‐1 levels (185.62 ± 56.28 µg/L, *p* = 0.037) and significantly lower ox‐LDL levels (448.24 ± 80.64 kU/L, *p* = 0.002), as shown in Table 3 and Figure 2.

**TABLE 3:** Comparison of PON‐1 and ox‐LDL levels between the two groups before and after therapy (¯*x* ± s).

Group	PON‐1 (µg/L)		ox‐LDL (kU/L)	
	Before	After	Before	After
Research (*n* = 50)	145.55 ± 50.40	185.62 ± 56.28	560.22 ± 72.48	448.24 ± 80.64
Control (*n* = 50)	149.20 ± 54.32	162.20 ± 54.24	562.80 ± 78.26	500.40 ± 85.20
*t*	0.348	2.119	0.171	3.144
*p*	0.729	0.037	0.865	0.002

### 3.4 Vascular Physiological Function of the Two Groups

**FIGURE 3:**
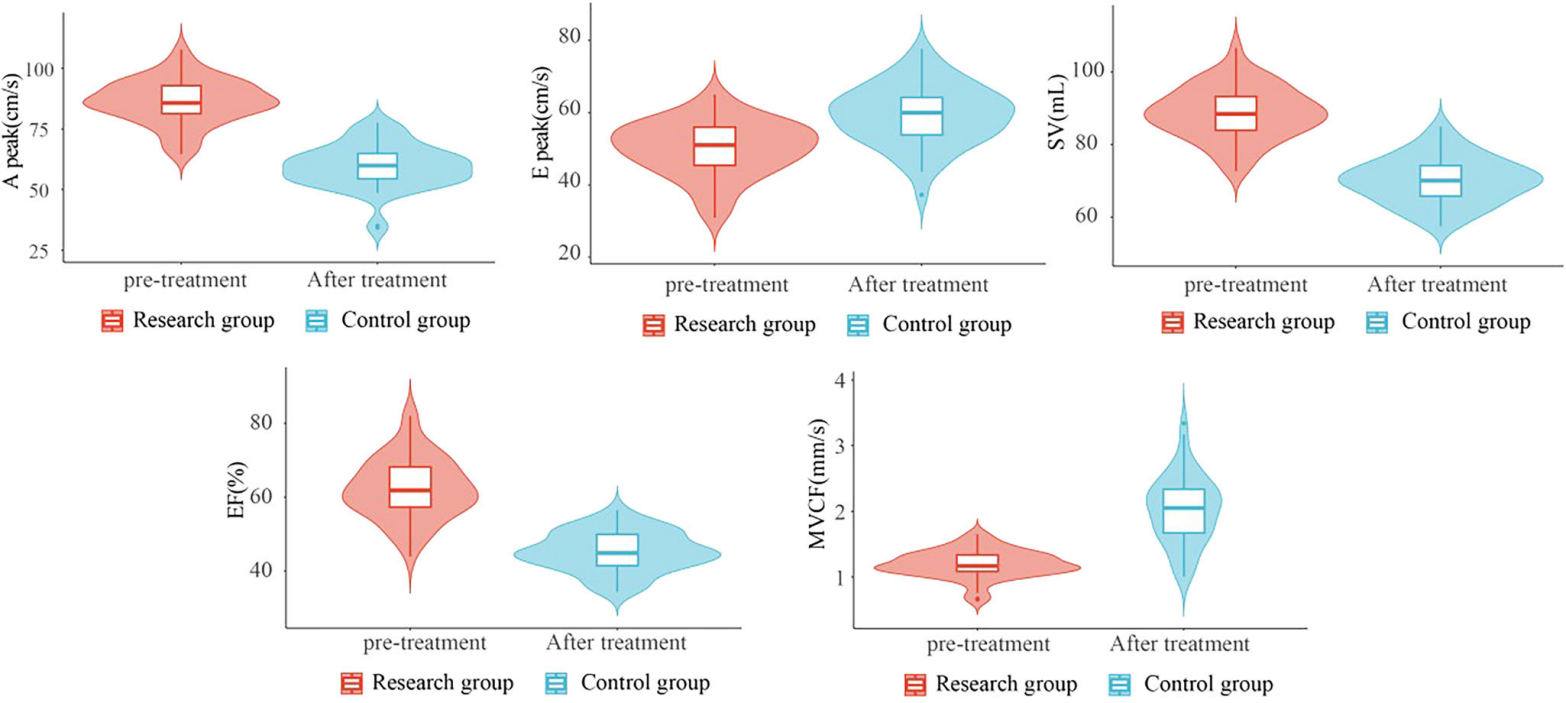
Comparison of vascular physiological function indicators between the two groups after therapy.

After treatment, compared with the control group [(59.62 ± 9.05) cm/s, (70.10 ± 6.35) mL, (45.20 ± 5.39)%, (59.40 ± 8.36) cm/s, (2.02 ± 0.52) mm/s], patients in the research group had significantly higher peak A (86.34 ± 9.22 cm/s), SV (88.64 ± 7.24 mL), and EF (62.32 ± 8.26%), and significantly lower peak E (50.10 ± 7.62 cm/s) and MVCF (1.20 ± 0.21 mm/s) (all *p* < 0.001), as shown in Table 4 and Figure 3.

**TABLE 4:** Comparison of vascular physiological function indicators between the two groups after therapy (¯*x* ± s).

Group	Peak A (cm/s)	Peak E (cm/s)	SV (mL)	EF (%)	MVCF (mm/s)
Research (*n* = 50)	86.34 ± 9.22	50.10 ± 7.62	88.64 ± 7.24	62.32 ± 8.26	1.20 ± 0.21
Control (*n* = 50)	59.62 ± 9.05	59.40 ± 8.36	70.10 ± 6.35	45.20 ± 5.39	2.02 ± 0.52
*t*	14.628	5.815	13.614	12.282	10.396
*p*	<0.001	<0.001	<0.001	<0.001	<0.001

### 3.5 Clinical Efficacy of the Two Groups

**FIGURE 4:**
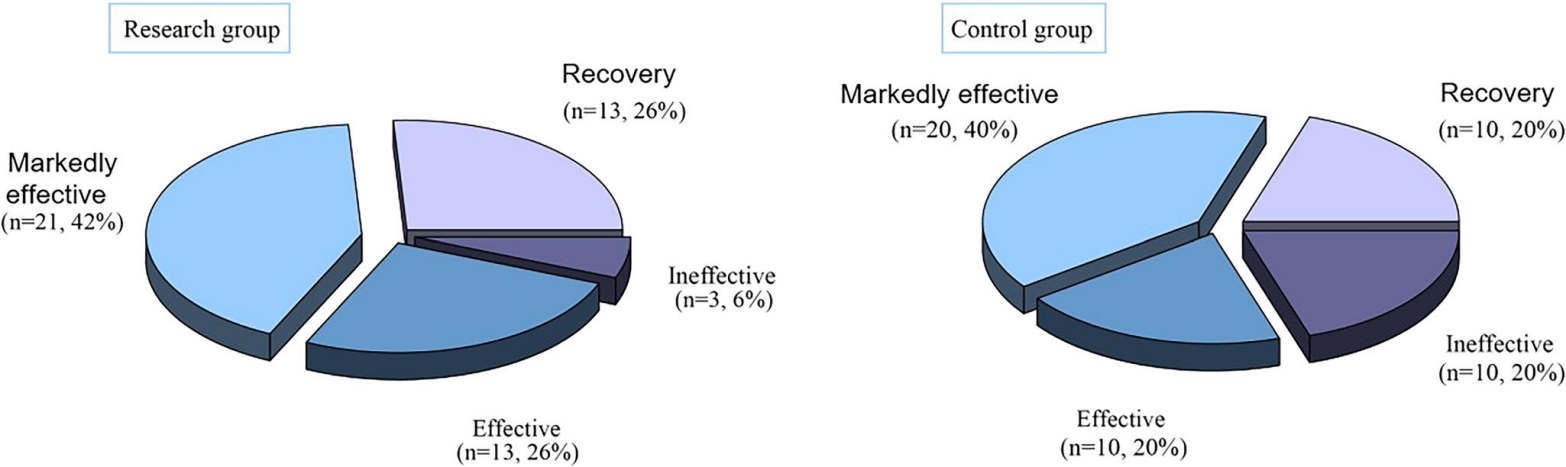
Comparison of clinical efficacy between the two groups.

The total effective rate was significantly higher in the research group (47 patients, 94.00%) than in the control group (41 patients, 82.00%) (*χ* ^2^ = 4.332, *p* = 0.037), as shown in Table 5 and Figure 4.

**TABLE 5:** Comparison of clinical efficacy between the two groups.

Group	Recovery	Markedly effective	Effective	Ineffective	Total effective rate
Research (*n* = 50)	13(26.00)	21(42.00)	13(26.00)	3(6.00)	47(94.00)
Control (*n* = 50)	10(20.00)	20(40.00)	10(20.00)	10(20.00)	41(82.00)
*χ* ^2^					4.332
*p*					0.037

### 3.6 Adverse Reactions of the Two Groups

**FIGURE 5:**
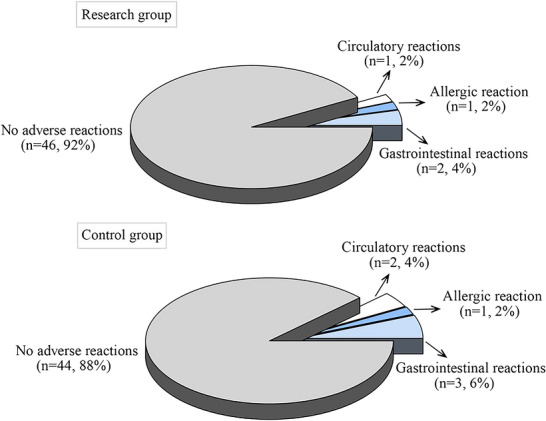
Comparison of adverse reactions between the two groups.

The incidence of adverse reactions was 8.00% (4 cases) in the research group and 12.00% (6 cases) in the control group. There was no statistically significant difference between the two groups (*χ* ^2^ = 0.444, *p* = 0.505), as shown in Table 6 and Figure 5.

**TABLE 6:** Comparison of adverse reactions between the two groups.

Group	Gastrointestinal reactions	Allergic reactions	Circulatory reactions	Overall incidence
Research (*n* = 50)	2(4.00)	1(2.00)	1(2.00)	4(8.00)
Control (*n* = 50)	3(6.00)	1(2.00)	2(4.00)	6(12.00)
*χ* ^2^				0.444
*p*				0.505

## 4 Discussion

Cerebral infarction is a very common critical illness in neurology, with an increasing incidence rate year after year, and atherosclerosis is the key pathological cause of the disease (Pedro‐Botet et al. 2020). The pathological process of ACI involves multiple aspects: abnormal proliferation of vascular smooth muscle cells leads to thickening of the vessel wall and stenosis of the vascular lumen; infiltration of inflammatory cells triggers a persistent inflammatory state that aggravates disease progression; and abnormal increase in extracellular matrix results in further thickening and hardening of the vessel wall (Wang et al. 2022; Zhang et al. 2022). Clinically, the disease is mainly treated by reducing blood lipids and inhibiting platelet aggregation (Cheng et al. 2020). Atorvastatin is an HMG‐CoA reductase inhibitor that reduces hepatic cholesterol synthesis, thereby lowering low‐density lipoprotein cholesterol levels, attenuating the degree of atherosclerosis, improving blood viscosity, and stabilizing atherosclerotic plaques (Xie et al. 2020; Tsuda et al. 2020).

As described in the Introduction, HPM combines moxibustion thermal stimulation with herbal pharmacological effects, allowing the medicinal components to be absorbed transdermally and distributed along the meridians to reach the diseased area, thereby fully exploiting the therapeutic effects of both acupoint stimulation and herbal medicine (Min et al. 2023). The current study employed a combination of atorvastatin and HPM to intervene in ACI, aiming to leverage their complementary mechanisms: the systemic anti‐atherosclerotic effect of atorvastatin and the local hemodynamic improvement from HPM.

In this study, the baseline clinical characteristics of the two groups were first compared, and the results confirmed that the groups were highly comparable in terms of age, sex, BMI, NIHSS score, and vascular risk factor profiles. Regarding the extent of atherosclerotic involvement beyond the cerebral vasculature, we found that carotid artery plaques were universal among enrolled patients (100.00%), intracranial arterial stenosis was present in 68.00%, and peripheral arterial disease was documented in 12.00%. These findings are consistent with the well‐established systemic nature of atherosclerosis (Song et al. 2020). Importantly, the distribution of multi‐territory atherosclerotic involvement was comparable between the two groups, minimizing potential confounding effects on the comparison of inflammatory and oxidative stress markers.

Studies have found that abnormal blood rheology can lead to increased circulatory resistance, increased vascular endothelial cell dysfunction, reduced microcirculatory perfusion, and cerebral hemodynamic disorders, thereby promoting the progression of cerebral infarction (Chen et al. 2022; Lee et al. 2022). The present study demonstrated that after therapy, the research group had significantly lower levels of all three hemorheological indicators compared with the control group. This suggests that atorvastatin combined with HPM is effective in improving blood flow status. Atorvastatin has been shown to not only exert anti‐inflammatory effects but also prevent thrombus generation by blocking the release of inflammatory factors from plaques and promoting the establishment of collateral circulation in the brain (Woźniak et al. 2023; Setiawan et al. 2023). As for the improvement mechanism of HPM, the herbal prescription used in this study (derived from Shaoyao Licorice Decoction and Buyang Huanwu Decoction) has the effects of promoting blood circulation and invigorating qi. Through the warming yang, dispersing cold, and activating collaterals action of moxibustion, HPM dilates blood vessels and accelerates blood flow, thereby improving hemorheological parameters (Hu et al. 2020; Zhou et al. 2021).

The pathogenesis of ACI is closely related to oxidative stress, which not only promotes the onset of the disease but also accelerates its progression. PON‐1 and ox‐LDL are biological markers of ischemic stroke associated with oxidative stress (Li et al. 2022; Basak et al. 2021). PON‐1 is a calcium ion‐dependent enzyme protein closely linked to high‐density lipoprotein, which hydrolyzes lipid peroxides and exerts antioxidant effects on low‐density lipoprotein, thereby reducing ox‐LDL levels and exerting anti‐atherosclerotic effects (Kimak et al. 2011; Hackenhaar et al. 2012). The present study found that after therapy, the research group had significantly higher PON‐1 levels and significantly lower ox‐LDL levels compared with the control group. This demonstrates that atorvastatin combined with HPM can inhibit the degradation of PON‐1, increase its activity, reduce ox‐LDL oxidation, and exert a synergistic antioxidant effect.

In addition, atherosclerosis can have serious adverse effects on blood vessels, including the coronary arteries, cerebral arteries, aorta, and peripheral vessels (Shang et al. 2023). Therefore, observation of vascular physiological function indicators is clinically important. The results of this study showed that the research group demonstrated superior vascular function parameters compared with the control group, including higher peak A, SV, and EF, and lower peak E and MVCF. This indicates that the combined treatment significantly improved vascular dilation, cardiac ejection function, cerebral blood supply, and overall blood circulation.

This study also observed the clinical efficacy of the two groups, and the total effective rate was significantly higher in the research group (94.00%) than in the control group (82.00%). This demonstrates that the combined treatment has higher efficacy and can improve clinical symptoms more effectively. Regarding safety, previous research has raised concerns about potential hepatotoxicity associated with statin therapy (Khan et al. 2020). In the present study, the incidence of adverse reactions did not differ significantly between the two groups (8.00% vs. 12.00%), and no cases of severe liver damage were observed, indicating that atorvastatin combined with HPM has an acceptable drug safety profile.

Several limitations of this study should be acknowledged. First, this was a single‐center study with a relatively small sample size of 100 patients, which may limit the generalizability of the findings. Second, due to the nature of moxibustion, blinding of patients and treating physicians was not feasible, although outcome assessors and the statistician were blinded. Third, the 12‐week treatment duration may not capture the long‐term effects and sustainability of the combined therapy. Fourth, we did not perform systematic screening for atherosclerosis in all arterial territories (e.g., abdominal aorta, renal arteries) in every patient, which may have led to underestimation of the systemic atherosclerotic burden. Future multi‐center, large‐sample, double‐blind studies with extended follow‐up periods and comprehensive vascular screening are warranted to confirm these findings and further elucidate the mechanisms underlying the synergistic effects of atorvastatin and HPM.

In summary, the treatment of patients with ACI with atorvastatin combined with HPM can improve oxidative stress and blood rheology by increasing PON‐1 activity and reducing ox‐LDL levels. The combined therapy demonstrates good clinical efficacy and is beneficial for patient prognosis.

## Author Contributions

**Wei An**: conceptualization, supervision. **Xinlei Hou**: investigation, methodology. **Ning Wei**: project administration, data curation. **Yuzhuo Zhou**: investigation, data curation. **Xinsheng Xie**: formal analysis. **Ze Jin**: formal analysis. **Linjing Wang**: writing – original draft. **Xuzhou Li**: writing – review and editing. **Weijia Zhuang**: methodology, resources. **Di Chen**: writing – review and editing, supervision.

## Funding

This work was supported by the Scientific research projects of Heilongjiang Provincial Health and Wellness Commission (Project No. 20232121020285).

## Ethics Statement

Approval for the study was obtained from the ethics committee of The Second Affiliated Hospital of Heilongjiang University of Traditional Chinese Medicine (No. IRB‐AF/SG‐21/02.0). All human studies reported in this article have been conducted in accordance with the ethical standards laid down in the 1964 Declaration of Helsinki and its later amendments. The study protocol was reviewed and approved by the appropriate ethics committee prior to the commencement of the research. Additionally, all relevant national laws and regulations concerning the ethical treatment of human subjects have been strictly observed.

## Consent

All patients provided written informed consent for participation in this study and for research authorization for record review. The study was approved by the institutional review board.

## Conflicts of Interest

The authors report there are no competing interests to declare.

## Supporting information




**Supplementary Materials**: brb371565‐sup‐0001‐CONSORT‐2025‐editable‐checklist.docx

## Data Availability

The individual participant data can be requested and accessed by contacting the study management teams of the studies on reasonable request.
